# G protein-coupled receptors mediate neural regulation of innate immune responses in *caenorhabditis elegans*

**DOI:** 10.14800/rci.1543

**Published:** 2017-05-24

**Authors:** Yiyong Liu, Jingru Sun

**Affiliations:** Department of Biomedical Sciences, Elson S. Floyd College of Medicine, Washington State University, Spokane, Washington, 99202, USA

**Keywords:** G protein-coupled receptors, OCTR-1, neural regulation, innate immunity, *C. elegans*

## Abstract

G protein-coupled receptors (GPCRs) are a large family of transmembrane proteins that perceive many extracellular signals and transduce them into cellular physiological responses. GPCRs regulate immunity in both vertebrates and invertebrates. However, the mechanisms responsible for such regulation are not fully understood. Recent research using the genetically tractable model organism *Caenorhabditis elegans* has led to the identification of specific GPCRs, neurotransmitters, neurons and non-neural cells in the regulation of innate immunity. Several neural circuits have been demonstrated to function in GPCR-dependent immuno-regulatory pathways. Besides being essential in neural-immune interactions, GPCRs also regulate innate immune response in non-neural tissues cell-autonomously through mechanisms independent of neural circuits. Here we review GPCR-mediated neural control of innate immunity in *C. elegans* and briefly discuss GPCR-dependent immune regulation via non-neural mechanisms.

## Introduction

G protein-coupled receptors (GPCRs) constitute the largest and most versatile superfamily of membrane bound signaling proteins. In *C. elegans*, about 7% of all predicted protein-coding genes encode approximately 1,300 GPCRs ^[1–3]^. In response to a diverse array of ligands/agonists, including neurotransmitters, peptides, large proteins, hormones, lipids, photons, and odorants, these receptors modulate key physiological functions such as neurotransmission, sensory perception, chemotaxis, and immune responses ^[4, 5]^. The basic architecture of GPCRs includes a seven-transmembrane domain, an extracellular N-terminal domain and an intracellular C-terminal domain. While these integral membrane proteins have relatively similar structural features in their intracellular part, large structural diversity appears in the extracellular part including the GPCR ligand-binding sites, which explains why these receptors recognize so many extracellular physical and chemical signals ^[5, 6]^. Binding of extracellular ligands to GPCRs induces conformational changes in the proteins, resulting in activation and release of the bound G protein in the cell. The signals then activate distinct downstream effectors that ultimately lead to physiological responses, usually via regulation of gene expression. GPCRs are regarded as potential therapeutic targets in many diseases with 50–60% of all marketed drugs targeting these receptors ^[7]^. Thus, it is not surprising that the 2012 Nobel Prize in Chemistry was awarded to Robert J. Lefkowitz of Duke University and Brian K. Kobilka of Stanford University for their studies of GPCRs (http://www.Nobelprize.org).

As GPCRs are capable of sensing extracellular stimuli and convert the stimuli into cellular responses, they function in vertebrates as mediators of signals involved in both innate and adaptive immunity ^[8]^. Recent studies in invertebrates, especially in the genetically tractable model organism *Caenorhabditis elegans*, identified specific GPCRs and neural cells in the control of immune responses ^[9–14]^, indicating that the immuno-regulatory function of GPCRs is evolutionarily conserved. *C. elegans* does not have an adaptive immune system and relies on innate immunity and avoidance behavior to defend itself against pathogenic attacks. GPCRs either function in non-neural tissues to control innate immune responses or act in the nervous system to modulate immunity in a cell non-autonomous manner. GPCRs are also involved in regulating pathogen aversive behavior. In this review, we focus on GPCR-mediated neural regulation of innate immunity in *C. elegans* and also briefly discuss GPCR-dependent immune control via non-neural mechanisms.

## *C. elegans* as a model system to study neural-immune communications

In nature, *C. elegans* lives in the soil and decaying organic matter, where it is in contact with various microbes. It eats innocuous bacteria as food sources and can be infected by a large number of pathogens including Gram-negative bacteria, Gram-positive bacteria, fungi and viruses. To date, more than 40 microbes have been shown to be pathogenic to *C. elegans*, some of which are also human pathogens ^[15]^. The major routes of infection are through the nematode’s intestine and epidermis. Because *C. elegans* does not have professional immune cells, intestinal and epidermal epithelial cells serve as the primary defense against pathogens, as they are in direct contact with the microbes. Despite lacking adaptive immunity, *C. elegans* can rapidly mount innate immune responses by triggering evolutionarily conserved signaling pathways ^[16–19]^. These pathways include the mitogen-activated protein kinase (MAPK) pathways, the DAF-2/insulin-like receptor pathway, the DBL-1 pathway (homologous to the mammalian TGF-β cascade), the unfolded protein response (UPR), and programmed cell death ^[15, 17–20]^. Activation of these cellular pathways induces the expression of immune effectors such as lectins, lysozymes, lipases, and antimicrobial peptides, which act directly or indirectly to combat the invading microbes ^[15, 16, 21]^.

Host innate immune responses to pathogen infection must be tightly regulated because insufficient responses exacerbate infection, whereas excessive responses lead to prolonged inflammation, tissue damage and death ^[22]^. A wealth of mammalian studies indicate that the nervous system plays a critical role in the regulation of immune responses ^[23, 24]^. Recent studies by us ^[10, 25, 26]^ and others ^[9, 11–14]^ have revealed that neural control of innate immunity in mammals has a homologous occurrence in *C. elegans*, one of the simplest organisms with a nervous system. This indicates that the regulatory mechanism dates back to the origins of the nervous system ^[27]^. Due to the complexity of the mammalian immune and nervous systems (an adult human brain contains about 86 billion neurons ^[28]^), it is difficult to dissect neural-immune interactions in mammals with current technology. *C. elegans*, by contrast, is an excellent model organism for such studies because of its simple, well-defined nervous system and an immune system that resembles the human innate immune system in several key respects ^[29, 30]^. *C. elegans* only has 302 neurons; the identity, morphology, and synaptic connectivity of each neuron are well described. It is the only animal for which the synaptic wiring diagram of the nervous system has been completely established ^[31]^. Also, most gene families involved in mammalian neuronal functions are found in *C. elegans* ^[32]^. Moreover, upon infection with microorganisms, including many human pathogens, *C. elegans* can mount innate immune responses by activating signaling pathways that are conserved in humans ^[33–35]^. Applying the *C. elegans* model system to study neural-immune signaling has greatly facilitated our understanding of neural-immune regulatory circuits ^[9–14, 25, 26]^.

## Octopamine receptor OCTR-1

**Figure 1:**
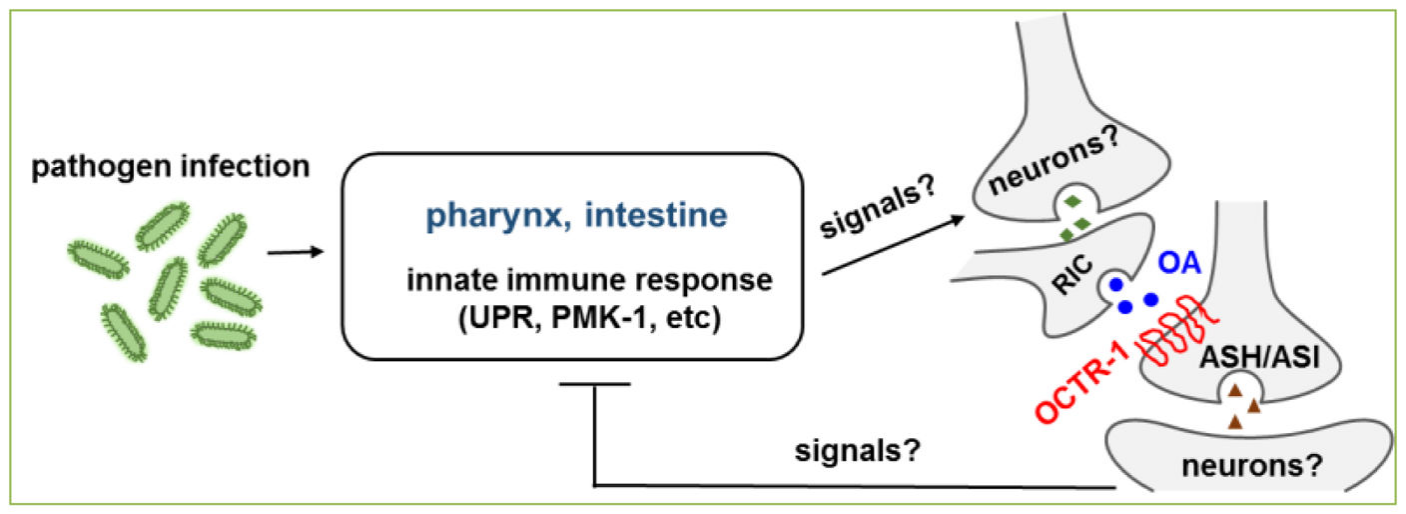
The octopaminergic immuno-inhibitory pathway in *C. elegans*.

*C. elegans* studies on neural-immune interactions have led to the identification of specific neuronal GPCRs and neural cells that regulate innate immunity ^[9–14]^. We demonstrated that *C. elegans* lacking OCTR -1, a octopamineGPCR, in two types of sensory neurons (designated as ASH and ASI) exhibited substantially improved survival against the human opportunistic pathogen *Pseudomonas aeruginosa* strain PA14 ^[10]^. This protection was not the result of enhanced pathogen avoidance or pathogen accumulation ^[10]^. Microarray and quantitative real time PCR (qRT-PCR) analyses showed that OCTR-1 suppresses the expression of non-canonical UPR genes of the *pqn/abu* family and genes in the p38/PMK-1 MAPK immune pathway ^[10]^. These genes are expressed predominantly in pharyngeal and/or intestinal tissues ^[10, 36, 37]^, indicating that ASH and ASI neurons in the head of *C. elegans* regulate innate immune responses in distant tissues cell non-autonomously (Figure 1). We further showed that neuronal OCTR-1 also regulates the canonical UPR pathway, which is controlled by the X-box binding protein 1 (XBP-1) ^[38, 39]^, at the organismal level ^[25]^. Importantly, XBP-1 is not under OCTR-1 control during development, only at the adult stage, indicating the nervous system temporally controls the UPR pathway to maintain endoplasmic reticulum (ER) homeostasis during development and immune activation ^[25]^. Recently we revealed that OCTR-1 regulates innate immunity at both the transcript and protein levels, and inhibits specific proteins synthesis factors such as ribosomal protein RPS-1 and translation initiation factor EIF-3.J to reduce infection-triggered protein synthesis and UPR ^[26]^.

OCTR-1 was initially identified as an octopamine receptor in behavioral responses of *C. elegans* to chemical stimulation ^[40]^. We found that octopamine is also an endogenous ligand of OCTR-1 in immune regulation (unpublished data). In *C. elegans*, octopamine is synthesized in two RIC interneurons and non-neuronal gonadal sheath cells ^[41]^. Our genetic ablation experiments indicate that RIC neurons function in OCTR-1-dependent innate immunity (unpublished data). These studies uncovered an octopaminergic immuno-inhibitory pathway in *C. elegans* that contributes to the maintenance of immunological homeostasis during pathogen infection (Figure 1): octopamine released from RIC neurons acts as a ligand of OCTR-1 in ASH and ASI neurons to suppress innate immune responses in pharyngeal and intestinal tissues. To further dissect OCTR-1-mediated neural regulation of innate immunity, additional work is needed to address questions such as which neurons form a circuit with RIC, ASH and ASI neurons in the OCTR-1 pathway, how *P. aeruginosa* infection activates the neural circuit, and the nature of the signals that are relayed from the nervous system to the non-neural tissues for immune regulation (Figure 1).

## Neuropeptide receptor NPR-1

**Figure 2:**
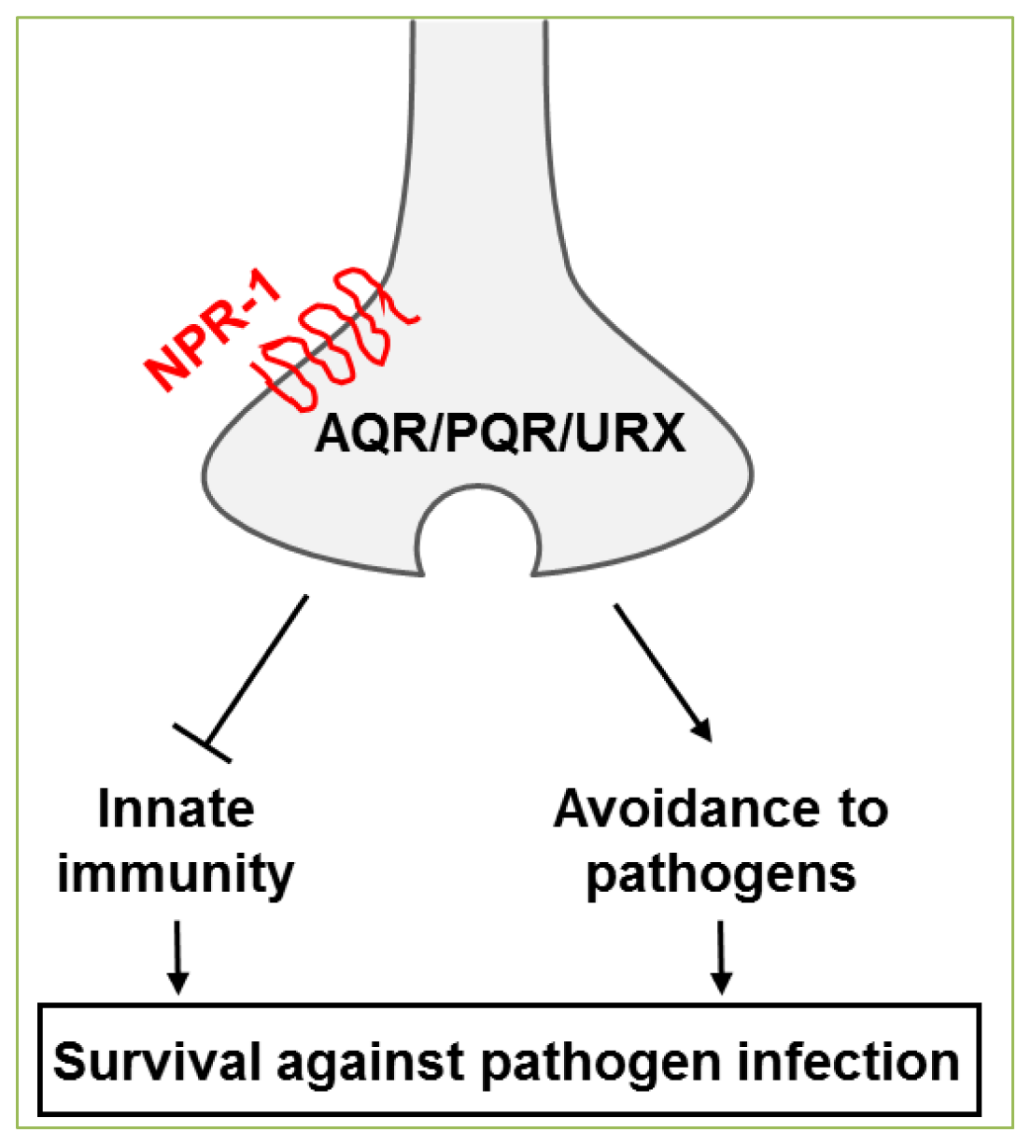
NPR-1 mediates neural regulation of innate immunity and avoidance behavior in response to pathogen infection (adapted from [65]).

NPR-1, a neuronal GPCR related to mammalian neuropeptide Y receptors, plays a direct role in immune regulation ^[12]^. *C. elegans* deficient in NPR-1 exhibits enhanced susceptibility to infections by *P. aeruginosa* and *Salmonella enterica* ^[12]^. Most of the genes that are misregulated in *npr-1(ad609)* animals (mutants with a loss-of-function allele of *npr-1*) correspond to markers of innate immune responses that are regulated by the DAF-2, DBL-1 or p38/PMK-1 MAPK signaling pathways ^[12]^. The expression patterns of immune genes differ over time between wild-type N2 animals and CB4856 animals carrying a polymorphism in *npr-1* ^[42]^. Neuron-specific rescue and genetic ablation experiments suggest that NPR-1 functions in AQR, PQR, and URX neurons to control immunity ^[12]^ (Figure 2). Recent studies also implicate an indirect role of NPR-1 in defense responses by regulating the nematode’s avoidance behavior to certain pathogens ^[8, 43–45]^. Animals carrying mutations in *npr-1* show a broad range of behavioral phenotypes ^[46]^, including a change in oxygen sensation ^[47, 48]^ that affect their ability to avoid pathogenic bacteria. NPR-1-mediated neural regulation of both immunity and avoidance behavior might contribute to the nematode’s overall survival against microbial attacks (Figure 2).

## Serotonin receptors SER-1 and SER-7

**Figure 3:**
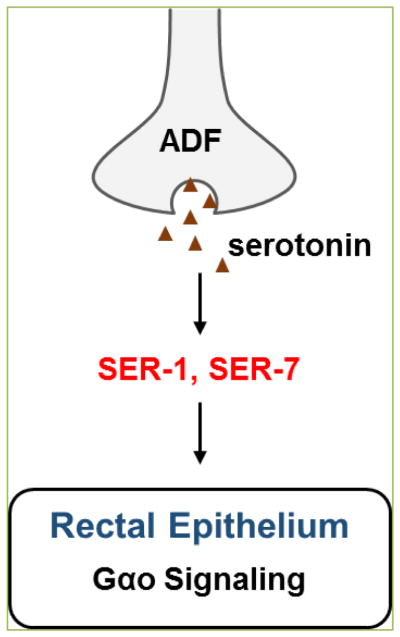
Serotonin receptors mediate neural regulation of innate immune responses in rectal epithelium (adapted from [66]).

Anderson *et al.* ^[9]^ demonstrated that upon infection of *C. elegans* with bacterial pathogen *Microbacterium nematophilum*, the neurotransmitter serotonin acts via its GPCRs SER-1 and SER-7 to suppress innate immune response in the rectal epithelium (Figure 3). In mammals, serotonin regulates both innate and adaptive immune responses ^[49, 50]^, suggesting that the immuno-regulatory function of serotonin is evolutionary conserved. Unlike in the context of *P. aeruginosa* infection, where serotonin signaling promotes aversive learning leading to behavioral pathogen avoidance ^[51]^, serotonin does not play a role in the avoidance response to *M. nematophilum* but suppresses the Deformed anal region (Dar) phenotype, a hallmark of immune response induced by *M. nematophilum* ^[52, 53]^. The suppression of Dar is mediated by SER-1 and SER-7 that activate the conserved G-protein signaling, GOA-1(Gαo) signaling, in epithelial cells ^[9]^. These receptors likely regulate the G-protein network in a cell non-autonomous manner because normally they are not expressed on rectal epithelial cells. However, pathogen infection may change their expression patterns. Serotonin synthesized in the amphid chemosensory neuron pair ADF by tryptophan hydroxylase (TPH-1), the rate-limiting enzyme in the biosynthesis of serotonin ^[54]^, is required for regulating innate immune responses to *M. nematophilum* ^[9]^. It is clear that ADF neurons in the animal’s head have the capacity to signal to distant rectal epithelial cells located in the animal’s tail (Figure 3). What is not clear, however, is how the immuno-modulatory signals are relayed from the neurons to non-neural tissues. Serotonin may function as a long-range signaling molecule to directly act on the distant cells. Alternatively, serotonin may activate SER-1 and SER-7 expressed on other neurons, which then release a signal to activate GOA-1 signaling in rectal epithelial cells. Nonetheless, the nature of the immuno-modulatory signals that are relayed from neurons to distal tissues remains to be determined.

## Dopamine receptor DOP-4

**Figure 4:**
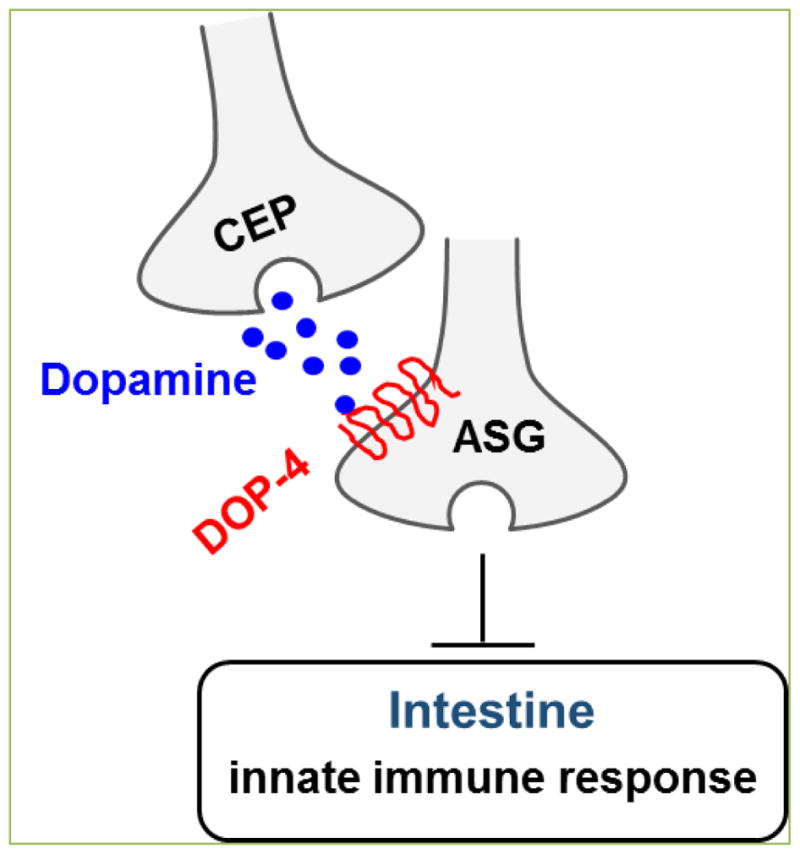
Proposed dopaminergic immuno-regulatory circuit (adapted from [11]).

Cao and Aballay ^[11]^ report that in *C. elegans*, dopamine signaling suppresses innate immune responses to *P. aeruginosa* infection by downregulating the p38/PMK-1 MAPK pathway. The immune suppression is mediated by DOP-4, a D1-like dopamine receptor. DOP-4 is expressed in various neurons, including ASG, AVL, and pharyngeal neurons, as well as in intestinal cells ^[55]^. The immuno-inhibitory function of dopamine originates in dopaminergic CEP neurons. Dopamine released from these neurons activates DOP-4 expressed in downstream ASG neurons. The dopamine signaling then suppresses innate immune responses in the intestine non-cell-autonomously by inhibiting the p38/PMK-1 MAPK signaling pathway. A putative dopaminergic immuno-regulatory circuit is depicted in Figure 4. In a separate study, Anyanful *et al.* ^[14]^ provided indirect evidence supporting the involvement of dopaminergic signaling in immune modulation. They reported that dopaminergic neurons and dopamine receptor DOP-3 are required for the conditioning of *C. elegans* to enteropathogenic *E. coli*, i.e. brief exposure of worms to the pathogen enhances their survival against a subsequent exposure that would otherwise prove lethal ^[14]^.

## GPCRs in innate immune responses to fungus *Drechmeria coniospora*

Regulation of *C. elegans* innate immune responses to its natural fungal pathogen *Drechmeria coniospora* involves both neural and non-neural mechanisms. Unlike many bacterium pathogens that establish infection through ingestion by *C. elegans*, conidia of *D. coniospora* attach to the nematode cuticle and penetrate the epidermis to initiate infection. Fungal infection elicits rapid innate immune responses in *C. elegans*, leading to the expression of antimicrobial peptide (AMP)-encoding genes, including genes of the *nlp* family and the *caenacin* family, in the epidermis ^[13, 56–58]^. While induction of the *nlp* genes is primarily dependent on the p38/PMK-1 MAPK pathway ^[56, 57]^, Zugasti and Ewbank found that upregulation of the *caenacin* genes is regulated by a noncanonical TGF-β signaling pathway ^[13]^. Neuron-derived ligand DBL-1 (a homolog of polypeptide TGF-β) acts in a non-cell-autonomous way to promote *caenacin* gene expression in the epidermis ^[13]^. However, the receptor for DBL-1 has not been identified, and the mechanisms responsible for this neural regulation of immunity remain unclear. The Ewbank group further demonstrated that DCAR-1, a GPCR initially identified as the receptor for repellent dihydrocaffeic acid (DHCA) in a *C. elegans* behavior study ^[59]^, is required for the *nlp* arm of the innate immune responses to *D. coniospora* infection ^[58]^. DCAR-1 is expressed on the apical surface in the main epidermal syncytium hyp7 as well as in the *C. elegans* nervous system including ASH, ASI and PVQ neurons ^[58, 59]^. Site-specific knockdown and rescue experiments indicate that DCAR-1 controls the expression of the *nlp* genes in the epidermis in a cell-autonomous manner, through an immuno-regulatory mechanism independent of neural circuits. HPLA (4-hydroxyphenyllactic acid), a structural isomer of DHCA, was identified as an endogenous ligand of DCAR-1 ^[58]^. The ligand/receptor pair HPLA/DCAR-1 was proposed to function as a sensor of damage-associated molecular patterns (DAMPs, host biomolecules that signal tissue damage) and trigger innate immune responses ^[58, 60]^.

## Regulation of innate immunity by GPCR through non-neural mechanisms

As illustrated above by DCAR-1-dependent immune response to *D. coniospora* infection, GPCRs also regulate innate immunity through non-neural mechanisms. Another example of GPCRs that mediate non-neural control of innate immunity is the *C. elegans* FSHR-1, a homolog of the follicle stimulating hormone receptor. FSHR-1 is expressed in several somatic tissues, most strongly in the intestine and neurons ^[61, 62]^. It acts in the intestine to regulate the nematode’s immune responses to diverse pathogens ^[63]^ and defense responses to heavy metals and oxidative stress ^[64]^. These cell-autonomous regulatory pathways add another layer of control that GPCRs have over innate immunity, which helps *C. elegans* maintain immunological homeostasis during pathogen infection.

## Concluding remarks

Use of *C. elegans* model system to study neural-immune signaling has identified specific GPCRs, neurotransmitters and neural cells in the regulation of innate immunity ^[9–14]^. Several neural circuits have been demonstrated to function in GPCR-dependent immuno-regulatory pathways, including an octopaminergic circuit ^[10]^, a serotoninergic circuit ^[9]^, and a dopaminergic circuit ^[11]^. Besides playing essential roles in neural control of immunity, GPCRs also mediate cell-autonomous regulation of innate immune response in non-neural tissues through mechanisms independent of neural circuits ^[58, 63, 64]^. These findings have greatly advanced our understanding of the molecular and cellular mechanisms responsible for maintaining immunological homeostasis during pathogen infection. Despite these significant advancements, many questions, however, remain unanswered. For example, how does pathogen infection activate the nervous system? How does the innate immune system reciprocally regulate the nervous system? Why are distinct neural circuits activated in response to different pathogens? What is the nature of the signals that are relayed from the nervous system to the non-neural tissues for immune regulation? Because the mammalian immune and nervous systems are highly complex and difficult to dissect at the molecular and cellular levels, *C. elegans* will remain to be a powerful model system for answering these questions.

## Abbreviations

GPCR: G protein-coupled receptor
OCTR-1: octopamine receptor
NPR-1: neuropeptide Y receptor
SER-1: 5-HT2 metabotripic serotonin receptor
SER-7: 5-HT7 metabotripic serotonin receptor
DOP-4: dopamine receptor D4
DHCA: dihydrocaffeic acid
HPLA: 4-hydroxyphenyllactic acid
FSHR-1: follicle stimulating hormone receptor
